# Prenatal Aspiration of Fetal Ovarian Cysts: When to Intervene? A Case Report and Review of the Literature

**DOI:** 10.1055/a-2562-1898

**Published:** 2025-04-10

**Authors:** Giulia Bonanni, Scott A. Shainker, Eyal Krispin, Ryne A. Didier, Terry L. Buchmiller, Alireza A. Shamshirsaz

**Affiliations:** 1 Division of Fetal Medicine and Surgery, Fetal Care and Surgery Center, Boston Children's Hospital, Harvard Medical School, Boston, Massachusetts; 2 Department of Women, Children, and Public Health Sciences, IRCCS Agostino Gemelli University Polyclinic Foundation, Catholic University of the Sacred Heart, Rome, Italy; 3 Department of Obstetrics and Gynecology, Beth Israel Deaconess Medical Center, Harvard Medical School, Boston, Massachusetts; 4 Department of Obstetrics, Gynecology, and Reproductive Biology, Harvard Medical School, Boston, Massachusetts; 5 Department of Radiology, Boston Children's Hospital, Harvard Medical School, Boston, Massachusetts; 6 Department of Surgery, Boston Children's Hospital, Boston, Massachusetts

**Keywords:** fetal adnexal cyst, ultrasound-guided aspiration, fetal intervention, individualized prenatal care, prenatal diagnosis

## Abstract

Fetal adnexal cysts present unique challenges during pregnancy, requiring careful management strategies to mitigate risks throughout gestation and delivery. We present the case of a 35-year-old G4P2 patient, referred to our center for a large adnexal cyst confirmed by ultrasound (US) and fetal MRI, with a calculated volume of 210 mL. Given the cyst's size and the family's strong preference for vaginal delivery (VD), US-guided aspiration was performed at 35 ^6/7^ weeks, followed by an uncomplicated spontaneous VD at 37 ^2/7^ weeks. Two weeks postpartum, the ovarian cyst re-accumulated, requiring laparoscopic-assisted cystectomy in a torsed but viable left ovary. This case demonstrates the importance of individualized prenatal care, where clinical decisions balance parental preferences with medical risks. Maximizing the opportunity for vaginal birth was a top priority for the family, and the successful reduction of the cyst's size through percutaneous aspiration minimized the risk of abdominal dystocia and allowed for a safe VD. We review relevant literature, emphasizing the need for further research to refine fetal intervention criteria and improve outcomes for such cases.

Advancements in ultrasound (US) technology have notably broadened the scope of fetal interventions, transforming diagnostic and therapeutic approaches for conditions identified throughout pregnancy. Among these conditions, the prenatal management of large fetal ovarian cysts remains controversial, as no controlled trials have been performed, and the prognostic implications of cyst US characteristics—such as size, septations, and wall thickness—are not yet fully understood. 1 2 Most fetal ovarian cysts are diagnosed by US in the third trimester, 3 with a reported false-positive rate of 7.5% (95% confidence interval [CI]: 4.4–11.4%). 3 4 Although nearly always benign, these cysts carry an increased risk of complications, such as ovarian torsion or hemorrhage, which rises as their diameter increase. 4 5 Higher rates of complications have been reported in cysts measuring ≥4.0 cm. 4 These risks highlight the potential role of prenatal treatment in reducing cyst size and averting complications. The criteria for in-utero US-guided aspiration remain debatable, with suggested interventions centering on cyst characteristics such as echogenicity, size, progression, fetal positioning, and gestational age at the time of consideration. 6

In this context, we present a case of a large fetal adnexal cyst prenatally treated with cyst aspiration and review the related literature. We discuss relevant clinical aspects, including the importance of multidisciplinary evaluation and shared decision-making.

## Case Report

A 35-year-old healthy Caucasian female, G4P2, with a spontaneous singleton pregnancy, was referred to our fetal care center at 33 weeks of gestation due to a fetal adnexal cyst in a female fetus. The US at 33 ^1/7^ weeks revealed an enlarged fetal abdominal circumference (>99th percentile), a central anechoic and avascular cyst in the fetal pelvis measuring 7.0 cm × 6.4 cm × 5.5 cm, and polyhydramnios with a maximum vertical pocket (MVP) of 10 cm.

**Fig. 1:**
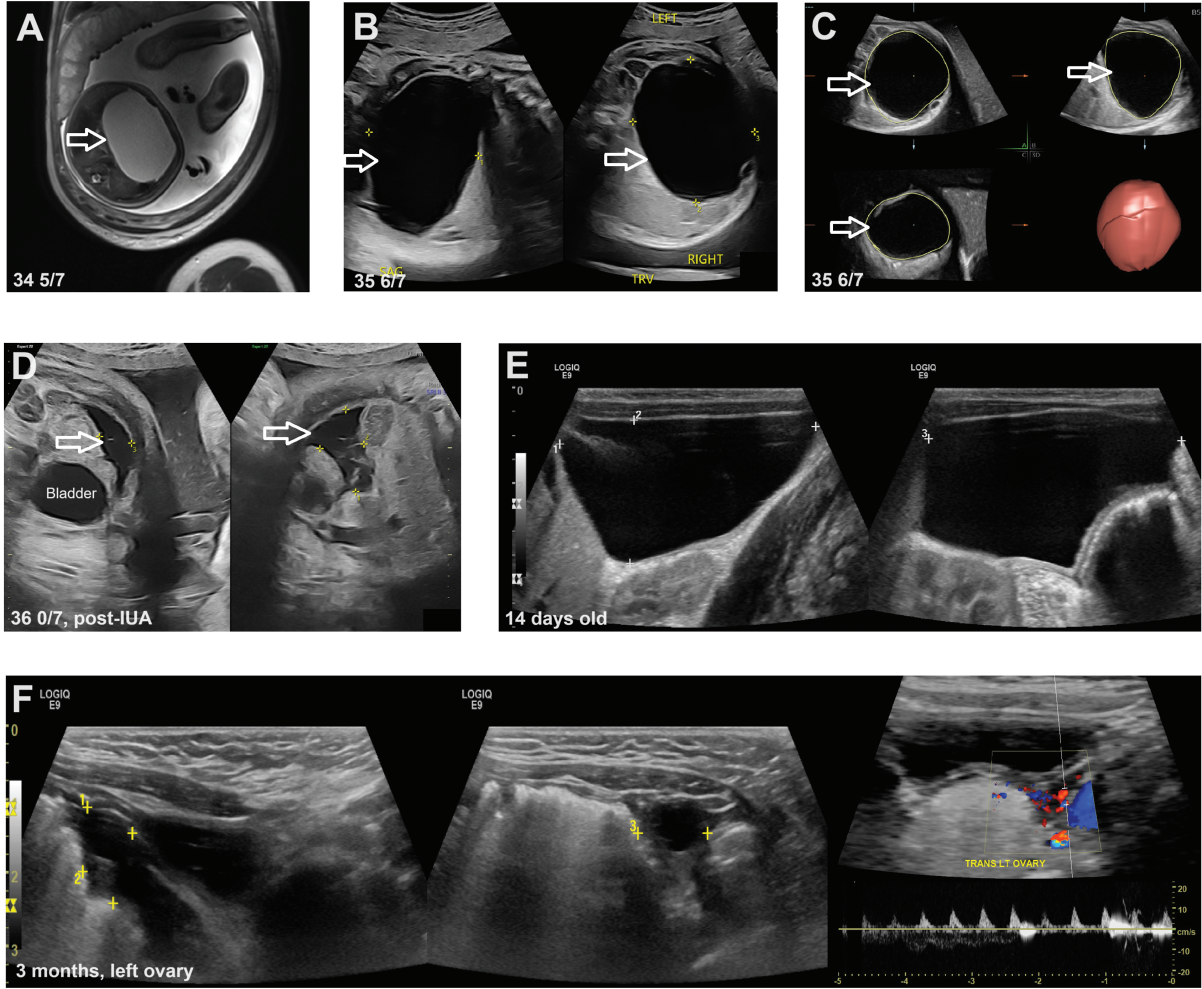
( **A** ) MRI at 34 ^5/7^ weeks showing a cyst suspected to be of ovarian origin, measuring 7.0 cm × 6.9 cm × 8.1 cm (arrow). ( **B, C** ) Follow-up ultrasound at 35 ^6/7^ weeks showed cyst enlargement (calculated volume > 210 mL; arrow) and associated polyhydramnios (AFI 27.8 cm, MVP 11.9 cm). ( **D** ) Limited transabdominal follow-up ultrasound at 36 ^0/7^ weeks revealing a small volume of free fluid in the fetal left lower quadrant (arrow) and absence of the cyst, with a high normal AFI of 21.1 cm and MVP of 6.7 cm. ( **E** ) Follow-up ultrasound at 14 days old showing a large, amorphous, anechoic cystic structure in the right pelvis, extending to the inferior hepatic margin (5.9 cm × 3.8 cm × 3.3 cm) with a single daughter cyst noted along the periphery of the lesion. No vascularity was observed. ( **F** ) Ultrasound at 3 months old showing normal appearance of the left ovary, containing a small follicle, with symmetric arterial flow and good arterial flow.

At 34 ^5/7^ weeks, MRI confirmed a cyst measuring 7.0 cm × 6.9 cm × 8.1 cm, suggestive of an ovarian origin ( Fig. 1A ). The US-calculated transverse abdominal diameter (TAD) was 11.2 cm. Management and delivery options, including in utero US-guided aspiration followed by vaginal delivery (VD), expectant management with VD, or elective cesarean delivery (CD), was thoroughly discussed with the maternal–fetal medicine specialists.

By 35 ^6/7^ weeks, the cyst had enlarged, with a calculated volume above 210 mL ( Fig. 1B and C ) and persistent polyhydramnios with an amniotic-fluid index (AFI) of 27.8 cm and an MVP of 11.9 cm. The patient emphasized her desire to maximize her chances of a vaginal birth and minimize neonatal interventions. Following multidisciplinary consultations, which included input from the neonatologist and the social worker, in utero US-guided aspiration was performed. Under local anesthesia, after fetal immobilization using fentanyl (35 mcg), atropine (70 mcg), and rocuronium (6.8 mg), a 20-gauge needle was used to aspirate 230 mL from the cyst and 800 mL of amniotic fluid, completely reducing the cyst's volume with no postoperative complications. A 12 mL sample of straw-colored, translucent fluid from the cyst was sent for cytological analysis, which showed a sparsely cellular specimen with no epithelial elements. A limited follow-up US the next day showed the cyst was no longer visible ( Fig. 1D ). The AFI decreased from 27.8 to 21.1 cm, and MVP from 11.9 to 6.7 cm.

Uncomplicated spontaneous VD occurred at 37 ^2/7^ weeks at an outside hospital, with a birth weight of 3,960 g. The neonate was discharged in excellent condition. At the 2-week visit with the pediatric surgeon, the mother reported the infant's increased fussiness postfeeding but no signs of gastrointestinal obstructive symptoms and normal weight gain. A mildly rounded abdomen was observed but the cyst was not palpable. A pelvic US revealed a large anechoic cyst (5.9 cm × 3.8 cm × 3.3 cm) in the pelvis extending to the level of the inferior hepatic margin, without evidence of torsion ( Fig. 1E ). A normal right ovary was seen. Given the cyst's size, both serial US surveillance and diagnostic laparoscopy with ovarian cystectomy (if the ovary was viable) versus oophorectomy (if not viable) were offered. The parents opted for surgery to maximize ovarian preservation.

At 20 days old, the infant underwent diagnostic laparoscopy, which confirmed a torsed but viable left ovary containing a large cyst consistent with the dimensions observed in US. A very small cyst was also identified on the normal right ovary. The left ovarian cyst was laparoscopically aspirated to facilitate exteriorization, followed by an ovarian cystectomy with preservation of the ovary. Pathological analysis revealed nonspecific findings, suggesting the cyst could represent an atretic cystic follicle, a pseudocyst, or a benign inclusion cyst. The infant's postoperative course was uneventful, and she was discharged the following day. A follow-up pelvic US performed at 3 months of age demonstrated two normal ovaries in a healthy, normally developing infant ( Fig. 1F ).

## Discussion and Review of the Literature

This case demonstrates the importance of individualized care in perinatal medicine, with decisions guided by a combination of parental preferences and clinical risk factors. The parents' strong desire for VD and preservation of ovarian function played central roles in shaping the management plan. Following a collaborative decision-making process, in utero US-guided aspiration allowed for a safe and uncomplicated VD, as the patient desired. Postnatally, diagnostic laparoscopy was chosen over serial monitoring to resolve the ovarian cyst definitively, with successful ovarian preservation.

Managing large or complex cysts remains a challenging area of prenatal care. In utero US-guided aspiration of fetal cysts can reduce cyst size, thereby potentially preventing torsion and its complications, such as necrosis, adhesion formation, and potential loss of the ovary with impaired ovarian reserve. 2 Current indications for fetal intervention are evolving, particularly for larger simple cysts. While complex cysts and simple cysts <4.0 cm are generally managed with observation, 7 there remains controversy regarding the optimal management of larger simple cysts due to the risks associated with in-utero intervention. 8 9 Although the specific risks associated with US-guided aspiration of fetal cysts have not been extensively documented, they are likely comparable to those of other in-utero needle procedures, such as amniocentesis. These risks include miscarriage, estimated at 0.1 to 0.3%, as well as preterm premature rupture of membranes, occurring in 1 to 2% of cases. 10

A recent retrospective cohort published by Bloomfield et al found that, among 52 patients with antenatally diagnosed ovarian cysts, 10 (19%) had no cyst at their first postnatal US. 11 Resolution rates were significantly higher and faster for simple cysts compared to complex cysts (84% vs. 52%, *p* < 0.05). 11 Similarly, a 2017 meta-analysis and a 2020 retrospective cohort study both highlighted that complex cysts (those containing blood or a solid component on imaging) and simple cysts ≥4.0 cm in diameter are less likely to resolve spontaneously either during pregnancy or after birth compared to simpler or smaller cysts, respectively. 3 4 Despite these findings, neither cyst size nor complexity was found to predict ovarian torsion in the retrospective cohort, 3 while the meta-analysis identified an increased risk of ovarian torsion and postnatal surgery in cysts ≥4.0 cm. 4

A 2017 review of 92 case reports and series reported on 380 cysts (324 observed and 56 aspirated in utero) in 365 fetuses. 12 The authors found that prenatal torsion rates were lower in aspirated cysts (4%, 2/56) compared to cysts managed conservatively sized 3.0 to 3.9 cm (15%, *p* = 0.02), 4.0 to 4.9 cm (27%, *p* < 0.001), 5.0 to 5.9 cm (34%, *p* < 0.001) and 7.0 to 7.9 cm (21%, *p* = 0.02). 12 Aspirated cysts had also lower postnatal surgery rates (7% vs. 49%, *p* < 0.001). 12 In 2018, a multicenter, prospective, randomized trial showed that aspiration of cysts >3.0 cm increases in-utero involution and reduces oophorectomy rates due to torsion. 13 However, recurrence rates after in-utero aspiration are notable (37.9%; 95% CI: 14.8–64.3%), with postnatal diagnoses of ovarian torsion and intracystic hemorrhage in 10.8% (95% CI: 4.4–19.7%) and 12.8% (95% CI: 3.8–26.0%), respectively. 4

An important concern with large pelvic cysts is the potential for abdominal dystocia, which may necessitate CD to prioritize maternal and fetal safety. Several case reports document dystocia arising from abdominal masses, though these cases differ from ours. Examples include a fetal cystic hygroma (14 cm ×11 cm) that caused difficult extraction, 14 as well as a fetus with enlarged kidneys where a TAD of approximately 12 cm led to dystocia requiring continued expulsive efforts and gentle fetal traction. 15 Even in a recent case of bowel dilation, dystocia was reported despite factors typically favoring a VD, such as prior successful VD, the preterm status of the fetus, and the expected compressibility of the abdomen. 16 While dystocia has not been reported with fetal ovarian cysts, our case illustrates the importance of anticipating potential risks. The cyst measured 7.0 cm × 6.9 cm × 8.1 cm, with an estimated TAD of 11.2 cm at 35 ^5/7^ weeks, presenting only a potential risk of dystocia. The strong maternal preference for a safe VD was carefully balanced against this risk. Ultimately, our intervention minimized the possibility of abdominal dystocia, facilitating a successful VD.

Further research is essential to improve standards of care for fetal abdominal cysts. Establishing a registry for these cases could help develop standardized protocols and improve outcomes. Comparing fetal and neonatal outcomes between in-utero aspiration and expectant management, stratified by US cyst characteristics and size, is essential to establish standardized protocols. Additionally, advancements in diagnostic criteria and imaging modalities would enhance prenatal diagnostic accuracy, facilitating better clinical decision-making.

## Conclusion

The successful in-utero US-guided aspiration of a large fetal ovarian cyst, in this case, highlights the importance of personalized care in achieving optimal maternal and neonatal outcomes. By effectively reducing cyst size, in-utero aspiration may play a role in facilitating spontaneous vaginal birth, aligning with the priorities of this family. This highlights the need for future research to confirm whether such interventions consistently contribute to favorable delivery outcomes. This case underscores the advancements in fetal intervention, where a nuanced understanding of cyst characteristics, gestational timing, and parental preferences guides shared decision-making. Ongoing studies are essential to refine the indications for and enhance the efficacy of prenatal interventions for fetal abdominal cysts, ultimately improving care for affected families.
